# Protective Effects of the Standardized Extract of *Amburana cearensis* in an Experimental Model of Severe Asthma: Suppression of Airway Inflammation and Modulation of Neutrophil Function

**DOI:** 10.1155/adpp/7371746

**Published:** 2026-11-15

**Authors:** Hilmara Hélia de Sousa Amaral, Talita Magalhães Rocha, Emmanuel Vinicius Oliveira Araújo, Ana Bruna de Araújo, Antonio Marcos Esmeraldo Bezerra, Janaina Serra Azul Monteiro Evangelista, Juvenia Bezerra Fontenele, Márcia Valéria Pitombeira Ferreira, Luzia Kalyne Almeida Moreira Leal

**Affiliations:** ^1^ Centro de Estudos Farmacêuticos e Cosméticos Faculdade de Farmácia Odontologia e Enfermagem Departamento de Farmácia Universidade Federal do Ceará Fortaleza Brazil ufc.br; ^2^ Laboratório de Morfologia Experimental Comparada Faculdade de Medicina Veterinária Universidade Estadual do Ceará Fortaleza Brazil uece.br; ^3^ Departamento de Patologia e Medicina Legal Universidade Federal do Ceará Fortaleza Brazil ufc.br

**Keywords:** airway inflammation, *Amburana cearensis*, asthma, coumarin, neutrophils, vanillic acid

## Abstract

*Amburana cearensis* A.C. Smith (Fabaceae) is traditionally used in Brazilian medicine for the treatment of asthma; however, its effects in experimental models of asthma exacerbation remain poorly understood. This study evaluated the effects of a standardized dried extract of *A. cearensis* (DEAC) and its chemical constituents—coumarin (CM) and vanillic acid (VA)—on airway inflammation in a murine model of ovalbumin (OVA) + lipopolysaccharide (LPS)‐induced asthma exacerbation and on human neutrophil activation. DEAC was characterized by high‐performance liquid chromatography with photodiode array detection (HPLC‐PDA) (markers: CM, VA, and amburoside). BALB/c mice were sensitized and challenged with OVA + LPS and treated with DEAC (100 mg/kg) and CM. On Day 26, bronchoalveolar lavage fluid (BALF) and lungs were analyzed for total and differential leukocytes, cytokines interleukin‐4 (IL‐4) and tumor necrosis factor‐*α* (TNF‐*α*), and histopathological changes. The effects of DEAC, CM, and VA on phorbol 12‐myristate 13‐acetate (PMA)‐induced human neutrophil activation were assessed through myeloperoxidase release and reactive oxygen species (ROS) production by chemiluminescence. OVA + LPS challenge induced severe airway inflammation, characterized by increased leukocyte recruitment, predominance of neutrophils, elevated IL‐4 and TNF‐*α* levels, and mucus hypersecretion. DEAC markedly reduced total leukocyte accumulation, restored the mononuclear cell proportion, and decreased neutrophil and eosinophil infiltration, with effects comparable to dexamethasone. CM reduced neutrophil accumulation without affecting total leukocytes. Lung histology confirmed decreased inflammation and mucus production. DEAC also lowered IL‐4 and TNF‐*α* levels. DEAC, CM, and VA inhibited neutrophil degranulation and ROS generation without affecting cell viability. These findings demonstrate the anti‐inflammatory potential of *A. cearensis* and support its traditional use as an adjuvant therapy for asthma within Brazilian public phytotherapy programs.

## 1. Introduction

Asthma is a heterogeneous chronic inflammatory airway disease characterized by respiratory symptoms, such as wheezing, shortness of breath, chest tightness, and coughing, together with variable expiratory airflow limitation [1]. A relatively new and prominent phenotype is neutrophilic asthma, characterized by predominance of neutrophils in sputum and bronchoalveolar lavage fluid (BALF). This phenotype has been associated with corticosteroid resistance, increased risk of acute exacerbations, and accelerated decline in lung function [1–3]. Neutrophil accumulation in the airways plays an important role in the course of asthma through the synthesis and release of numerous inflammatory mediators, such as cytokines, chemokines, reactive oxygen species (ROS), and proteases [4, 5].

Given the many adverse effects of corticosteroids and the challenges in treating neutrophilic asthma, there is an urgent need to search for novel and safer therapeutic strategies. In this context, medicinal plants such as *Amburana cearensis* appear to be a promising source. In Brazil, this species has been extensively used in traditional medicine and is included in the public phytotherapy program called “Farmácias Vivas” as an adjuvant in the treatment of asthma [6–8].

A substantial body of data has been generated by our research group with *A. cearensis* (a tree from the Caatinga biome), including chemical characterization and reports of biological activities such as bronchodilator, neuroprotective, antinociceptive, and anti‐inflammatory effects [9–15]. However, due to the intensive exploitation for both traditional medicine and logging, this species is at risk of extinction [16]. To address this concern, our research group has conducted chemical and pharmacological studies with young specimens cultivated under agronomic control. Similar to wild plants, the main bioactive constituents of the cultivated species are coumarin (CM), vanillic acid (VA), and amburoside (AMB), which are responsible, at least in part, for its anti‐inflammatory activity [12, 17, 18].

Therefore, this study investigated the effects of the standardized dry extract of cultivated *A. cearensis* (DEAC) in an experimental model of acute asthma exacerbation (or acute severe asthma), as well as the effects of its active compounds, CM and VA, on neutrophil functions, to improve our understanding of the therapeutic potential of this species for asthma.

## 2. Materials and Methods

### 2.1. Botanical Material

*Amburana cearensis* (Fabaceae) was cultivated from seeds collected from native mother plants, located in the rural area of Quixeramobim, Ceará, Brazil (5°12′08″, 39° 17′11″). The aerial parts and xylopods of *A. cearensis* were collected after 7 months of cultivation. Exsiccates of the species (Nos. 52618 and 52619) are deposited in Prisco Bezerra Herbarium, Department of Biology, Federal University of Ceará.

### 2.2. Animals

Female BALB/c mice (20–25 g) were obtained from the Multidisciplinary Center for Biological Research in the Laboratory of Animal Science—CEMIB, of the State University of Campinas (UNICAMP). Animals were fed standard rodent chow and water *ad libitum*. Animal experiments were approved by the Ethics Committee on Research at the Federal University of Ceará (protocol: 12/2016).

### 2.3. Chemicals

The chemicals used were CM (≥ 98%), VA (≥ 97%), ovalbumin (OVA), lipopolysaccharide (LPS), aluminum hydroxide, phorbol‐12‐myristate‐13‐acetate (PMA), myeloperoxidase (MPO), indomethacin, dimethyl sulfoxide (DMSO), 5‐amino‐2,3‐dihydro‐1,4‐phthalazinedione, Triton X‐100, 3,3′,3,5′‐tetramethylbenzidine (TMB), periodic acid–Schiff (PAS) (Sigma‐Aldrich, USA), gelatin (Difco, USA), P.A. alcohol, trypan blue dye, fetal bovine serum (FBS) (Gibco by Life Technologies, USA), formaldehyde (Dinâmica, Brazil), hematoxylin and eosin (H&E) (Êxodo Científica, Brazil), hydrogen peroxide (Sinty, Brazil).

### 2.4. Production and Characterization of DEAC by HPLC

The ethanol extract of *A. cearensis* was obtained by cold maceration followed by tubolysis, and the dry extract was prepared by spray drying according to the methodology described by Lima [17]. The determination of bioactive markers, VA and CM, in the DEAC was performed using high‐performance liquid chromatography (HPLC) coupled with a diode array detector (DAD), Waters model 2996. The separations were carried out on an *X*‐Terra (Waters) analytical column, 250 mm in length and 4.6 mm in diameter, with octadecyl silica reverse phase (C18), filled with 5‐μm spherical particles. The mobile phase consisted of two solutions, HPLC grade acetonitrile (Phase A) and water (Phase B). HPLC‐DAD analysis of cultivated *A. cearensis* dry extract allowed the identification and quantification of VA and CM. Identification was confirmed by comparing the retention time of working standards (Sigma, USA) (Figure 1), and the levels found were as follows: 2.55 mg/g VA and 14.25 mg/g CM.

**FIGURE 1:**
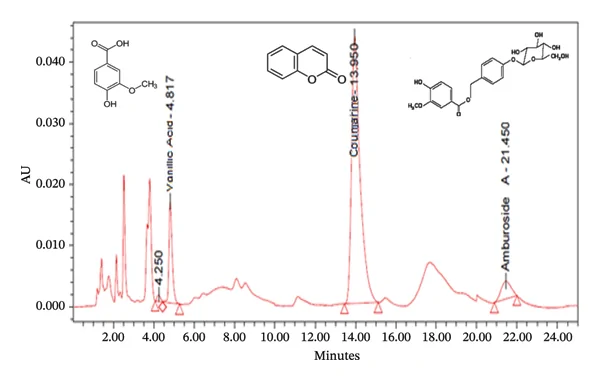
Chromatographic profile of dry extract from *Amburana cearensis* (DEAC) and identification of three compounds (vanillic acid, coumarin, and amburoside A).

### 2.5. Experimental Design

The experimental model of acute severe asthma was based on the protocol described in [19] with adaptations to OVA and LPS concentrations, stimulus interval period, and protocol duration. Sensitization and challenge conditions for BALB/c mice with OVA + LPS were standardized according to the accumulation of inflammatory cells in the BALF and histological evaluation of lung tissue, and the following experimental conditions were selected: animals were sensitized with LPS (2 μg/mL, i.p.) and, 30 min later, OVA (400 μg, adsorbed in 4 mg/mL aluminum hydroxide, i.p.) on the 1st and 8th days of the experiment. The challenges occurred for 6 consecutive days, from the 20th to the 25th day of the experiment. The animals received LPS (2 μg/mL), and 30 min later, they were exposed to 10% OVA nebulization for 30 min. We administered the respective treatments 30 min before each LPS challenge, once daily from Day 20 to Day 25. For the challenge, mice were placed into a plastic chamber (20 × 30 × 21 cm) attached to a nebulizer.

### 2.6. Evaluation of the Anti‐Inflammatory Activity of DEAC in an Acute Asthma Exacerbation Model

To evaluate the effects of DEAC on inflammatory parameters in an experimental model of asthma exacerbation, the animals were divided into the following groups: sham (animals sensitized and challenged with saline and sterile PBS, respectively); control (vehicle‐treated animals, DMSO 2% i.p., sensitized and challenged with LPS and OVA), and treatment groups receiving dexamethasone (DEXA, 1 mg/kg, i.p.), DEAC (100 mg/kg, i.p.), or CM (10 mg/kg, i.p.). All treatment groups were sensitized and challenged with LPS and OVA, as described previously.

Twenty‐four hours after the final challenge, the animals were anesthetized, and tracheal cannulation was performed via surgical exposure to collect BALF. A 0.5‐mL volume of sterile, ice‐cold PBS was instilled into the lungs via the tracheal cannula using a 1‐mL syringe, and the PBS was recovered by gentle aspiration.

The BALF and lung tissue samples were collected to determine inflammatory markers and histopathological analysis. The collected samples were used partially for total and differential leukocyte counts, and the remainder for cytokine levels and histopathological analysis.

#### 2.6.1. Total and Differential Leukocyte Count in Bronchoalveolar Lavage

An aliquot of the BALF was diluted with a 1:1 Turk solution for the total leukocyte count in a Neubauer chamber. The remainder of the recovered wash was centrifuged for 10 min at 800 × *g* and 4°C. The supernatant was stored for further analysis, and the cell pellet was resuspended in PBS with 10% FBS for differential leukocyte count. Counting slides were prepared by cytocentrifugation (500 rpm for 5 min) and stained with Panoptic rapid stain kit. The cells were examined under a light microscope using a 100× objective. One hundred leukocytes were counted and, based on their morphological aspects and staining, were divided into three groups: mononuclear cells, eosinophils, and neutrophils.

#### 2.6.2. Histopathological Analysis

After lung removal, the right lobe of each animal was fixed in 10% buffered formaldehyde (v/v) and, after 48 h, transferred to 70% (v/v) ethanol. Tissue was embedded in paraffin, and 4‐μm‐thick histological sections were obtained and stained with H&E for visualization of inflammatory cell infiltration or with PAS for visualization of mucus.

#### 2.6.3. Immunoassay (Enzyme‐Linked Immunosorbent Assay [ELISA])

The supernatant of BALF, obtained after centrifugation (800 × *g*, 10 min, 4°C), was used for cytokine quantification by ELISA, as previously described [20]. Interleukin‐4 (IL‐4) and tumor necrosis factor alpha (TNF‐α) levels were measured using mouse‐specific commercial kits (BD Biosciences, San Jose, CA, USA), according to the manufacturer’s instructions.

### 2.7. Evaluation of Anti‐Inflammatory Activity of DEAC, CM, and VA in Primary Neutrophil Culture

#### 2.7.1. Primary Polymorphonuclear (PMN) Cell Culture

Polymorphonuclear cells, predominantly neutrophils (80%–90%), were isolated from human blood provided by the Ceará Hemotherapy and Hematology Center‐HEMOCE (buffy coat samples). All protocols used were approved by the Human Research Ethics Committee of the Federal University of Ceará, protocol No. CAAE 45668515.4.0000.5054. The blood was centrifuged at 800 × *g* for 10 min; the plasma was discarded, and the cellular portion was treated with saline containing 2.5% (w/v) gelatin to separate blood components and yield a neutrophil‐enriched suspension [21]. Residual erythrocytes were lysed using 0.83% ammonium chloride (NH_4_Cl), followed by centrifugation and washing with saline. We assessed the final leukocyte preparation by leukocyte counting with Türk’s solution and confirmed neutrophil predominance (80%–90%) based on nuclear morphology.

#### 2.7.2. MTT Assay

Neutrophils (5 × 10^6^ cells/mL) were exposed to DEAC, CM, and VA (1, 10, 25, 50, and 100 μg/mL), Hanks’ Balanced Salt Solution (HBSS—untreated group), 1% (v/v) DMSO (control), or 0.2% Triton X‐100 for 30 min at 37°C and then incubated with MTT for 3 h. DMSO was then added to solubilize the formazan product. The absorbance was measured at 550 nm [22].

#### 2.7.3. Neutrophil Degranulation

Neutrophil suspension (5 × 10^6^ cells/mL) was incubated for 30 min at 37°C with DEAC, CM, and VA (1, 10, 25, 50, and 100 μg/mL), DMSO (control), Hanks’ solution (untreated cells), or indomethacin (100 μg/mL, standard drug). Then, PMA (0.1 μM) was added for 15 min at 37°C. The reaction was stopped by cooling, the suspension was centrifuged (2000 × *g*, 10 min, 4°C), and the supernatant obtained was used to determine the MPO concentration according to the methodology described by Úbeda et al. [23].

#### 2.7.4. ROS Production

Neutrophils (5 × 10^6^ cells/mL) were incubated at 37°C for 20 min with DEAC, CM, or VA (1, 10, 25, 50, and 100 μg/mL) and with the chemiluminescent probe luminol (280 μM). After the incubation period, the stimulus (PMA 0.1 μM) was added, and the production of ROS was monitored for 40 min at 37°C in a microplate reader. In all tests, spontaneous ROS production by cells in the absence of stimulation was measured. Quercetin (50 μg/mL) was used as a standard drug. The results were expressed as a percentage of ROS production in relation to the control.

### 2.8. Statistical Analysis

Results are expressed as the mean ± SEM of at least three independent experiments. Group differences were analyzed using Student’s *t* test for comparisons between two means, or analysis of variance (ANOVA), where the significance of the contrasts between the means was studied by the Tukey test. The significance level was set at *p* < 0.05.

## 3. Results

### 3.1. Evaluation of the Effect of DEAC in an Experimental Model of Exacerbated LPS + OVA‐Induced Asthma in BALB/c Mice

The standardization of the asthma exacerbation model allowed us to determine the conditions of sensitization and challenge of the animals. Animals were sensitized and challenged for 25 days, and on Day 26, showed an increase in leukocytes (23.58 ± 2.5 × 10^4^ cells/mL), predominantly neutrophils (36.2 ± 3.3%), compared with the unchallenged group (leukocytes: 3.92 ± 0.5 × 10^4^ cells/mL; neutrophils 11.25 ± 2.3%) (Figure 2A,B). In addition, the histological analysis showed the presence of mucus in the sensitized animals challenged with OVA + LPS (Figure 2C).

**FIGURE 2:**
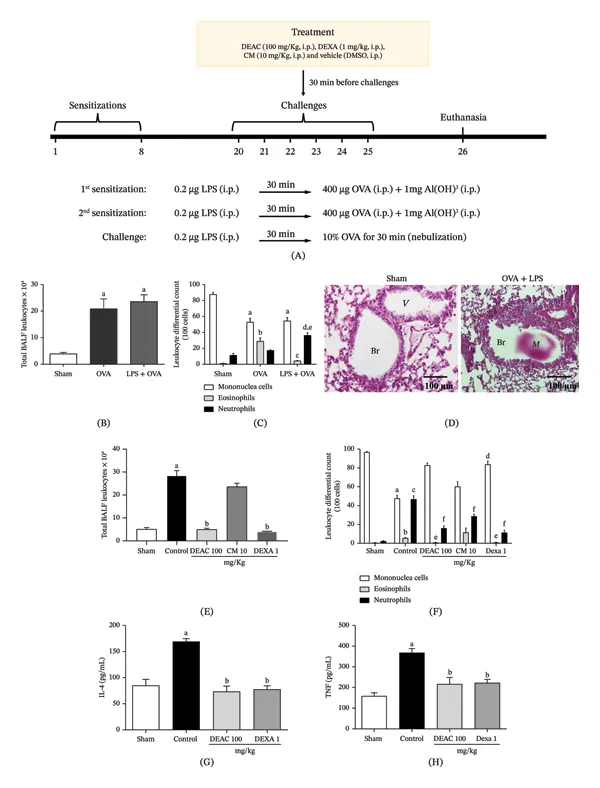
Effect of DEAC and CM on LPS + OVA‐induced pulmonary inflammation in BALB/c mice. Figure 2A illustrates the experimental timeline and treatment schedule. Total and differential leukocyte counts, as well as cytokine (IL‐4 and TNF‐*α*) levels, were determined in BALF collected 24 h after the last challenge. Animals (*n* = 7) were treated with vehicle (2% DMSO), DEAC (100 mg/kg, i.p.), CM (10 mg/kg, i.p.), or dexamethasone (DEXA) (1 mg/kg, i.p.) 30 min before each challenge; the sham group received sterile PBS. Representative lung tissue sections stained with periodic acid–Schiff (PAS) showing mucus secretion and cellular infiltration (D). Images were obtained at 40× magnification. Br, bronchioles; V, blood vessels; and M, mucus secretion. Results are expressed as mean ± SEM. Statistical analysis was performed using one‐way ANOVA followed by Tukey’s post hoc test. For graph B: a vs. sham group. For graph C: a vs. sham group (mononuclear cells); b vs. sham group (eosinophils); d vs. OVA group (mononuclear cells); e vs. OVA group (eosinophils). For graph E: a vs. sham group; b vs. control group. For graph F: a vs. sham group (mononuclear cells); b vs. sham group (eosinophils); c vs. control group (neutrophils); d vs. OVA group (mononuclear cells); e vs. OVA group (eosinophils); f vs. control group (neutrophils). For graphs G and H: a vs. sham group; b vs. OVA group.

The effect of DEAC on BALF total leukocyte accumulation in animals challenged with LPS + OVA can be seen in Figure 2D. The challenge of animals with LPS + OVA (control group) promoted a significant increase in the number of BALF total leukocytes (28.5 ± 2.4 × 10^4^ cells/mL) compared to the sham group (5.11 ± 0.65 × 10^4^ cells/mL). Treatment of animals with DEAC (100 mg/kg, i.p.) reduced by approximately 80% the number of cells present in BALF (5.0 ± 0.5 × 10^4^ cells/mL) compared to the control group, thus inhibiting BALF leukocyte accumulation similar to the standard drug DEXA (1 mg/kg, i.p.), which reduced cellularity by 85% (4.11 ± 0.4 × 10^4^ cells/mL).

The challenge of animals with LPS + OVA promoted the predominance of neutrophils over the eosinophil population among the accumulated BALF leukocytes, and a reduction in mononuclear cells in the inflammatory focus (control group [LPS + OVA]: 47.7 ± 3.2%) compared to the sham group (96.8 ± 0.6%). Treatment with DEAC (100 mg/kg, i.p.: 82.8 ± 2.2%) and DEXA (1 mg/kg, i.p.: 83.7 ± 3.3%) reestablished BALF mononuclear cell numbers. Regarding eosinophil cell migration in the BALF of BALB/c mice, it can be observed that the challenge with LPS + OVA increased cell migration (5.5 ± 0.6%) compared to the sham group (0.5 ± 0.2%). However, DEAC (100 mg/kg, i.p.: 0.7 ± 0,3%) and DEXA (1 mg/kg, i.p.: 0.8 ± 0.4%) significantly reduced the number of eosinophils at the inflammation site compared to the control group. Regarding neutrophils, it was observed that there was a significant increase in the migration of these cells to BALF (47.0 ± 3.5%) in the control group compared to the sham group (2.3 ± 0.6%), and for this cell population, the treatment of animals with DEAC (100 mg/kg, i.p.) and CM (10 mg/kg, i.p.) significantly reduced neutrophil migration to the BALF (16.0 ± 2.4% and 28.6 ± 2.5%, respectively), compared to the control group (Figure 2E).

The results obtained showed that DEAC administered intraperitoneally at 100 mg/kg (i.p.) in animals challenged with OVA + LPS exhibited an anti‐inflammatory response, inhibiting the accumulation of inflammatory cells in BALF and producing results comparable to those observed in animals treated with DEXA (1 mg/kg, i.p.: 11.2 ± 2.5%).

### 3.2. Effect of DEAC on Cytokine Levels in Bronchoalveolar Lavage of Animals Challenged by LPS + OVA

The challenge of animals with LPS + OVA increased approximately 100% the concentration of IL‐4 in BALF (control group: 169.22 ± 5.6 pg/mL) compared to the sham group (85.1 ± 11.8 pg/mL). Treatment of the animals with DEAC (100 mg/kg, i.p.) as well as with DEXA (1 mg/kg, i.p.) significantly reduced IL‐4 levels (DEAC: 73.48 ± 10.24 pg/mL; DEXA: 77.5 ± 6.8 pg/mL) (Figure 2F).

Regarding the levels of TNF‐α in BALF of animals challenged with OVA + LPS, it was also observed that the challenge provided an intense inflammatory response, expressed by the significant increase in TNF‐α concentration (367.9 ± 20.6 pg/mL) compared to the sham group (158.8 ± 16.12 pg/mL). Similar to DEXA (222.8 ± 16.67 pg/mL), DEAC (217.0 ± 31.68 pg/mL) provided a significant reduction in TNF‐*α* when compared to the control group. These reductions corresponded to 39.5% (Figure 2G).

### 3.3. Histopathological Analysis of Lung Tissue From LPS + OVA‐Challenged BALB/c Mice Treated With DEAC

The histopathological analysis of the lung tissue of the animals (Figure 3) showed that the challenge with LPS + OVA caused intense cellularity in the peribronchial and perivascular region (Figure 3B) when compared to the sham group (Figure 3A), and in the bronchioles (Figure 3B) compared to the sham group (Figure 3A). The treatment with DEAC (100 mg/kg, i.p.) showed lower cellularity, and no mucus production was observed (Figure 3C). Similar results were observed in the DEXA group (1 mg/kg, i.p.) (standard drug) (Figure 3D).

**FIGURE 3:**
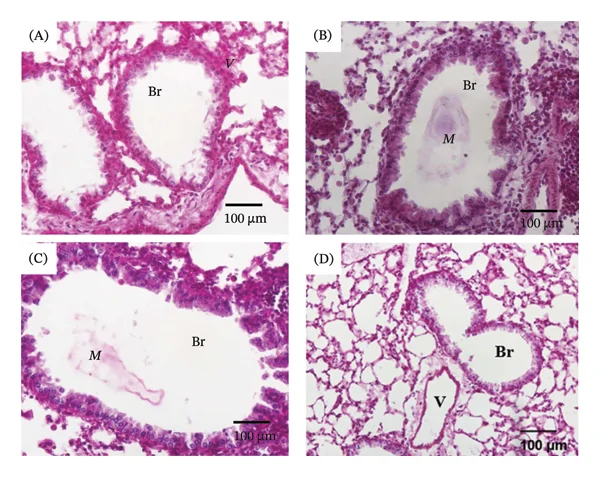
Effects of DEAC, or dexamethasone treatment on lung tissue of LPS + OVA‐challenged BALB/c mice. Analysis of mucus presence in PAS‐stained tissue at 40× magnification. Br indicates bronchioles; V, vessels; and M, mucus secretion. Groups: Sham–A; Control–B; DEAC (100 mg/kg, i.p.)–C; and dexamethasone (1 mg/kg, i.p.)–D.

### 3.4. Effect of DEAC, CM, and VA on PMA‐Induced Degranulation Measured by the Enzyme MPO

Before initiating the MPO assay, cell viability was assessed using the MTT assay. Neither DEAC nor its active compounds (VA and CM) caused a reduction in cell viability at concentrations of 1–100 μg/mL compared to the HBSS group (100% viable). The effects of DEAC, CM, and VA (1, 10, 25, 50, and 100 μg/mL) on neutrophil degranulation are shown in Figure 4. The addition of DEAC to the neutrophil suspension significantly inhibited PMA‐induced MPO release, with a maximum inhibition of 42.3 ± 2.9% at 100 μg/mL in MPO secretion compared to the control group. Standard anti‐inflammatory indomethacin (36 μg/mL) inhibited neutrophil MPO release by 73.3 ± 1.8% (Figure 4A). In Figure 4B, CM reduced the release of MPO from 10 μg/mL (59.9 ± 9.3%), inhibiting the release of this enzyme by up to 80.2 ± 2.8% at 100 μg/mL, an effect comparable to that of indomethacin (63.3 ± 2.8%). VA significantly reduced the release of MPO from 10 μg/mL. The effect size at a concentration of 100 μg/mL was comparable to that of indomethacin (87.7 ± 1.0% [VA] and 84.8 ± 1.0% [indomethacin], respectively) (Figure 4C).

**FIGURE 4:**
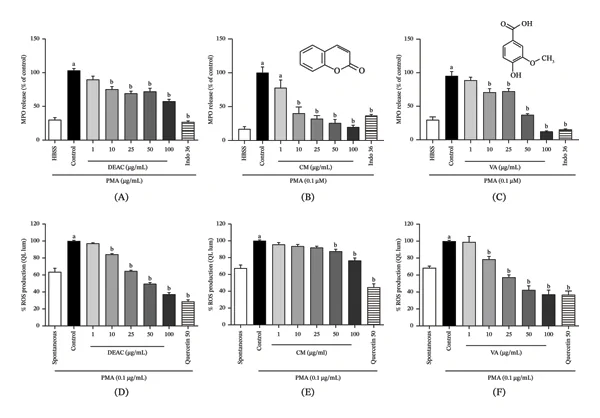
Effect of DEAC, CM, and VA on PMA‐stimulated neutrophils. Neutrophils (2.5 × 10^6^ cells/mL) were incubated with DEAC, CM, or VA (1–100 μg/mL) and stimulated with PMA (0.1 μM). Degranulation was assessed by MPO release and ROS generation by luminol chemiluminescence. HBSS: unstimulated cells; Control: vehicle (1% DMSO); INDO: indomethacin (36 μg/mL); quercetin (50 μg/mL). Results are expressed as mean ± SEM. Statistical analysis was performed using one‐way ANOVA followed by Tukey’s post hoc test. a vs. spontaneous; b vs. control; c vs. 1 (DEAC, CM, or VA); d vs. 10 (DEAC, CM, or VA); e vs. 25 (DEAC, CM, or VA); f vs. 50 (DEAC, CM, or VA); g vs. 100 (DEAC, CM, or VA); h vs. INDO 36 or quercetin 50.

### 3.5. Effect of DEAC, CM, and VA on the Production of ROS

The antioxidant activity of DEAC, CM, and VA (1, 10, 25, 50, and 100 μg/mL) was evaluated by chemiluminescence using the luminol probe. The addition of DEAC in increasing concentrations to the neutrophil suspension significantly reduced the production of ROS in a concentration dependent manner, with a maximum inhibition of 62.72% at the highest concentration. Quercetin (50 μg/mL), an antioxidant standard, reduced the production of ROS by 71.26% (Figure 4D). The treatment of cells with CM, however, caused a discrete but significant reduction of 23.4% in the production of ROS marked by luminol (Figure 4E). The addition of VA to the suspension of neutrophils promoted a significant reduction in the production of ROS marked by luminol from 10 μg/mL, achieving up to 62.82% inhibition (Figure 4E).

## 4. Discussion

This study demonstrated that cultivated *A. cearensis* exerts significant protective effects in an experimental model of asthma exacerbation. The standardized dried extract of *A. cearensis* (active markers: CM, VA, and AMB) reduced airway leukocyte infiltration, inhibited mucus hypersecretion, decreased cytokine levels, and modulated neutrophil activation.

Asthmatic patients, particularly those predisposed to more severe phenotypes, display enhanced neutrophil chemotaxis, which may occur even in the absence of acute allergen exposure. This intrinsic susceptibility is further amplified after bronchial challenge and is associated with elevated levels of cytokines in serum and sputum [24]. This highlights the central role of neutrophils in severe and treatment‐refractory asthma phenotypes [25]. In our murine model of LPS + OVA‐induced asthma exacerbation, neutrophils represented the predominant inflammatory cell type in BALF, closely reflecting the neutrophilic profile observed in human severe asthma [25]. Thus, this model provides a translationally relevant platform to investigate therapeutic strategies targeting neutrophil‐driven inflammation. The use of LPS in association with OVA, in addition to aluminum hydroxide as an adjuvant, is well recognized to induce a more robust Th2/Th17 response, leading to the establishment of asthma exacerbation in rodents [26, 27].

The administration of DEAC markedly attenuated airway inflammation induced by OVA + LPS, producing effects comparable to DEXA, the reference anti‐inflammatory drug. Future studies will evaluate different doses of DEAC to establish a dose–response relationship and investigate other routes of administration, including oral administration, to elucidate its pharmacological profile. The reduction in total leukocyte counts observed after treatment with DEAC appears to be primarily due to the inhibition of neutrophil recruitment, which represents the predominant cell type in this asthma exacerbation model. Notably, DEAC, like DEXA, restored the relative proportion of mononuclear cells in BALF, suggesting a selective effect against excessive neutrophilic infiltration while preserving, or even favoring, regulatory and adaptive immune cell populations. Such a shift in the cellular profile may be critical for controlling airway inflammation without completely suppressing protective immune functions, thereby contributing to a more balanced resolution of the inflammatory response. A similar result was observed when animals were treated with CM, the active ingredient of DEAC. These data support the anti‐inflammatory effect of *A. cearensis*, corroborated by our previous reports on the plant extract and its main bioactive constituents (CM, VA, and AMB) [11–13, 27]. Ratifying the role of chemical constituents in the anti‐inflammatory effect of *A. cearensis*, we previously determined the antiedematogenic activity of both the plant extract, CM, and VA in the carrageenan‐induced mice paw edema. This effect was associated with the presence of both CM and VA, which inhibited the paw edema and the leukocyte migration in the rat peritoneal cavity induced by carrageenan. Furthermore, studies with AMB have shown its ability to inhibit carrageenan, prostaglandin E_2_‐ or histamine‐induced paw edema, and to reduce neutrophil migration in a carrageenan‐induced peritonitis model [12, 28].

Preclinical translation in asthma research is strengthened by combining in vivo animal models with complementary in vitro assays using human cells. In BALB/c mice, combining OVA sensitization and challenge with low‐dose LPS shifts the classic Th2‐driven allergic response toward a Th17‐associated, neutrophilic airway endotype, allowing the evaluation of inflammatory cell infiltration and cytokines such as IL‐17, TNF‐*α*, and IL‐6 in BALF. Complementing these findings with in vitro functional assays using human neutrophils, including MPO degranulation and luminol‐amplified ROS production, provides translational support by demonstrating that the effects observed in the murine model are also relevant to human neutrophil function. Together, these findings support the potential relevance of DEAC for severe neutrophilic or mixed granulocytic asthma endotype, while further studies using corticosteroid‐resistant models are needed to determine its potential in corticosteroid‐refractory disease [29, 30].

Many immune cells and structural cells have a key role in asthma pathogenesis. When activated, these cells release several chemical mediators, including cytokines, such as TNF‐α and IL‐4, which contribute to the airway inflammation and remodeling in asthma [31, 32]. Recent studies [33, 34] confirm that targeting the TNF‐*α* and IL‐4 pathways remains a promising strategy for severe asthma management. The TNF‐α cytokine is strongly associated with the recruitment of neutrophils and eosinophils and corticosteroid resistance, while IL‐4 regulates Th2 differentiation and eosinophil recruitment and immunoglobulin class switching [28, 31]. Thus, a key observation in our study was the reduction of both TNF‐α and IL‐4 levels in BALF by DEAC.

Histological analysis of the lung tissue from LPS + OVA‐challenged BALB/c treated with DEAC showed that the plant extract suppressed inflammatory cell migration and inhibited mucus hypersecretion, similar to DEXA. Taken together, these data suggest that DEAC possibly mitigates mucus production partially through inhibition of neutrophil infiltration and IL‐4‐driven goblet cell hyperplasia.

Considering that neutrophilic activation and degranulation play a pivotal role in the pathogenesis of severe and corticosteroid‐resistant asthma, the study continued evaluating the effect of DEAC and its main constituents, CM and VA, on human neutrophil degranulation. DEAC, CM, and VA significantly reduced PMA‐induced neutrophil degranulation measured by MPO release, but CM (CE_50_: 6.8 μg/mL) was more potent than VA (CE_50_: 33.5 μg/mL). MPO is one of the main enzymes released by neutrophils and is involved in the asthmatic process. Studies have detected the presence of high MPO levels in the BALF and sputum of asthmatic patients [35]. PMA is a diacylglycerol analog (DAG) that activates signal transduction in neutrophils, acting directly on protein kinase C (PKC). PKC is responsible for triggering the release of Ca^2+^ from intracellular reserves, which increases its concentration inside the cell, thus causing degranulation [36]. Therefore, it is suggested that DEAC and its bioactive compounds, VA and CM, not only reduce the accumulation of inflammatory cells, such as neutrophils, in the inflammatory focus but also appear to modulate the activation of these cells without affecting cell viability.

Overproduction of ROS from infiltrating immune cells, particularly eosinophils and neutrophils, leads to oxidative stress in asthma, causing cellular damage [37]. DEAC, VA, and CM reduced ROS production, as measured using the luminol chemiluminescent probe. Luminol can detect intra‐ and extracellularly generated ROS, especially those derived from the MPO‐H_2_O_2_‐HOCl system [38]. Further studies are warranted to elucidate the molecular mechanisms underlying these effects, particularly the involvement of redox‐sensitive signaling pathways such as NF‐κB and MAPKs.

Taken together, our findings indicate that DEAC exerts multitarget protective effects in asthma exacerbation, including suppression of airway leukocyte accumulation, inhibition of Th2 cytokine production, reduction of mucus hypersecretion, and modulation of neutrophil activation and ROS production. These results highlight the therapeutic potential of *A. cearensis* as a promising phytomedicine for the management of severe asthma phenotypes poorly responsive to corticosteroids.

The limitation of this study is that only a single dose of DEAC was tested in vivo. In addition, it is not clear how the in vitro results obtained with concentrations of up to 100 μg/mL correlate with the findings observed in the in vivo model. Further pharmacokinetic and tissue‐distribution studies are warranted to determine the systemic and airway exposure to DEAC and its bioactive constituents and to establish the translational relevance of the concentrations used in vitro. Therefore, future studies should evaluate dose–response relationships of the standardized *A. cearensis* extract administered via the oral route, in order to improve its pharmacological characterization and translational relevance. In addition, further investigations are needed to elucidate the involvement of key inflammatory signaling pathways, particularly MAPKs and NF‐κB, and to assess the effects of both the extract and its major constituents on these pathways. Such studies will contribute to a better understanding of the mechanisms underlying the observed anti‐inflammatory effects.

## 5. Conclusion

This study demonstrated that the standardized DEAC exerts significant anti‐inflammatory effects in an OVA + LPS‐induced asthma exacerbation model. Its protective activity was associated with reduced leukocyte infiltration—particularly neutrophils and eosinophils—suppression of IL‐4 and TNF‐*α* production, and modulation of neutrophil activation through inhibition of degranulation. These findings highlight the therapeutic potential of DEAC as a promising phytomedicine for severe asthma, while also indicating that its effects may be partly mediated by bioactive constituents such as CM and VA.

## Author Contributions

Hilmara Hélia de Sousa Amaral: writing–original draft, formal analysis, and methodology. Talita Magalhães Rocha: writing–original draft, methodology. Emmanuel Vinicius Oliveira Araújo: methodology. Ana Bruna de Araújo: writing–original draft and writing–review and editing. Antonio Marcos Esmeraldo Bezerra: methodology. Janaina Serra Azul Monteiro Evangelista: conceptualization, formal analysis, and methodology. Juvenia Bezerra Fontenele: methodology and conceptualization. Luzia Kalyne Almeida Moreira Leal: writing–review and editing, conceptualization, formal analysis, writing–original draft, and acquisition of financing. Márcia Valéria Pitombeira Ferreira: methodology.

## Funding

L.K.A.M. Leal acknowledges the Conselho Nacional de Desenvolvimento Científico e Tecnológico (CNPq) for their research fellowships (316948/2021‐7). This work was supported by the Coordenação de Aperfeiçoamento de Pessoal de Nível Superior (CAPES), the Fundação Cearense de Apoio ao Desenvolvimento Científico e Tecnológico (FUNCAP), and CNPq.

## Conflicts of Interest

The authors declare no conflicts of interest.

## Data Availability

The data that support the findings of this study are openly available in UFC Institutional Repository at http://www.repositorio.ufc.br/handle/riufc/28570.
